# Anger, Aggression and Violence: Towards an integrative, ‘transdiagnostic’ treatment paradigm

**DOI:** 10.1192/j.eurpsy.2025.1676

**Published:** 2025-08-26

**Authors:** S. Anand, S. Kapoor, S. Rizvi, R. Varanasi, Z. Abbas, I. Arora, H. Chhatwal

**Affiliations:** 1 Ashburn Psychological and Psychiatric Services, Ashburn, United States; 2 Windsor University School of Medicine, Cayon, Saint Kitts and Nevis; 3 Medics International, New Jersey; 4 Willis-Knighton Health System, Shreveport; 5 University of Texas - Houston, Houston; 6 SABA Unversity School of Medicine, Devens; 7 George Mason University, Fairfax, United States

## Abstract

**Introduction:**

Using a ‘transdiagnostic’ paradigm for psychopathology, we review the evidence-base for the different treatment interventions for anger, aggression and violence (AAV). We aim to more accurately link the range of psychopathology associated with AAV to underlying brain structures and functional neurocircuitry. Building upon these findings, we propose a working model that may have greater clinical utility than reliance upon a purely ‘disorder-based’ paradigm for addressing AAV. This will permit greater understanding of when anger (which has also conferred evolutionary survival advantages) can become maladaptive and destructive. This comprehensive, ‘interventionist’ model will hopefully lead to better clinical conceptualization of AAV, especially since most AAV presents more commonly in the context of interpersonal/situational stressors and to non-forensically trained clinicians.

**Objectives:**

1. To identify biological, cognitive, disorder-based, developmental and personality predispositions to AAV
2. To link neuroanatomical and functional brain circuitry with onset and maintainence of AAV
3. To identify *how* pharmacological and non pharmacological interventions work, and ‘where’ in the brain,
4. To review the evidence-base supporting various treatment interventions

**Methods:**

Database review of PUBMED, PsychINFO, Google Scholar and treatment guidelines dating back 15 years (review articles, treatment guidelines, meta-analyses and randomized controlled trials).

**Results:**

This will be visually depicted in an electronic slide (or >1 slide, if permitted) to identify:

1. The inter-relationship between contextal factors, the psychopathology of AAV and undelying brain ‘circutry’
2. The specific nature of the impulsive anger, ‘reactive’ aggression and violence response ‘cycle’ and where in this cycle various interventions can be effectively utlized
3. Which patient populations respond best to which types of interventions

**Conclusions:**

1. AAV are hyperarousal states, with outcomes that can be successfully managed
2. The evidence-base for the role of medications is more limited
3. The evidence-base for the role of various psychotherapies is greater, with specific therapies being particularly useful across psychiatric disorders and populations
4. Psychodynamic approches remain curently underutilized and underappreciated
5. Both medications and therapy influence brain neuroplasticity separately and synergistically

**Disclosure of Interest:**

None Declared

